# New synonym of *Tipula (Vestiplex) wahlgrenana* Alexander, 1968 (Diptera: Tipulidae)

**DOI:** 10.3897/BDJ.2.e4237

**Published:** 2014-12-30

**Authors:** Pavel Starkevich, Sigitas Podenas

**Affiliations:** † Department of Zoology, Vilnius University, Ciurlionio 21/27, LT-2009, Vilnius, Lithuania; ‡ Nature Research Centre, Akademijos Str. 2, LT-08412, Vilnius, Lithuania

**Keywords:** Crane flies, type material, hypopygium, India.

## Abstract

Based on examination of type specimens a crane fly species *Tipula (Vestiplex) hugueniniana* Alexander, 1971 is proposed as junior synonym of *Tipula (V.) wahlgrenana* Alexander, 1968. The new synonymy is based on similarity of male genitalia.

## Introduction

Females of the long-palped crane flies belonging to the subgenus *Tipula (Vestiplex)* Bezzi, 1924, are characterized by an ovipositor with the cerci powerfully constructed, heavily sclerotized with outer margins serrate, smooth in several Asiatic species; hypovalvae small to rudimentary (Alexander 1935, Alexander 1965, Alexander and Byers 1981). The male genital complex is extremely polymorphic (Savchenko 1964), usually with tergite 9 forming a shallowly concave sclerotized saucer; other species have their tergite 9 completely divided longitudinally by a pale membrane (Alexander 1934, Alexander 1935, Alexander and Byers 1981).

The world fauna of the the subgenus *Tipula (Vestiplex)* includes 170 valid species and subspecies level taxa, which are distributed throughout the Nearctic, Palaearctic and Oriental Regions (Oosterbroek 2014).

## Materials and methods

The type material used in this paper was obtained from the Smithsonian Institution, National Museum of Natural History, Washington, D. C. U.S.A. (USNM).

Specimens were studied with an Olympus SZ51 microscope. Pictures were taken with an Olympus SZX10, camera Evolution^TM^MP, program Immage-Pro Express 6.0. (Media Cybernetics).

Terminology of morphological features generally follows that of Alexander and Byers 1981.

## Taxon treatments

### Tipula (Vestiplex) wahlgrenana

Alexander, 1968

Tipula (Vestiplex) wahlgrenana Alexander 1968: 371, Plate 1, fig. 8; Plate 3, fig. 24.
Tipula (Vestiplex) wahlgrenana Alexander and Alexander 1973: 66.
Tipula (Vestiplex) hugueniniana Alexander 1971: 406, fig. 35. **syn. n.**

#### Materials

- **Type status:** Holotype. **Occurrence:** recordedBy: Schmid; sex: male; preparations: antennae, leg, wing and genitalia on slide mounted; **Taxon:** genus: Tipula; subgenus: Vestiplex; specificEpithet: wahlgrenana; scientificNameAuthorship: Alexander, 1968; **Location:** country: India; stateProvince: Kumaon; verbatimLocality: Khumyara, Pauri Garhwal; verbatimElevation: 4300-5000 feet [1311-1524 m]; **Event:** eventDate: 28 May 1958; **Record Level:** institutionCode: USNM
- **Type status:** Holotype. **Occurrence:** recordedBy: Schmid; sex: male; preparations: antenna, legs, wing and genitalia on slide mounted; **Taxon:** genus: Tipula; subgenus: Vestiplex; specificEpithet: hugueniniana; scientificNameAuthorship: Alexander, 1971; **Location:** country: India; stateProvince: Sikkim; verbatimLocality: Nanga; verbatimElevation: 5000 feet [1524 m]; **Event:** eventDate: 11 May 1959; **Record Level:** institutionCode: USNM
- **Type status:** Paratype. **Occurrence:** recordedBy: Schmid; sex: male; preparations: antenna, leg, wing and genitalia on slide mounted; **Taxon:** genus: Tipula; subgenus: Vestiplex; specificEpithet: hugueniniana; scientificNameAuthorship: Alexander, 1971; **Location:** country: India; stateProvince: Kumaon; verbatimLocality: Duldhar, Pauri Garhwal; verbatimElevation: 4500 feet [1372 m]; **Event:** eventDate: 2 June 1958; **Record Level:** institutionCode: USNM

## Discussion

According to the Catalogue of the Craneflies of the World (Oosterbroek 2014), there are currently 36 species of crane flies belonging to the the subgenus *Tipula (Vestiplex)* recorded in India. Three species were described by Edwards (Edwards 1927, Edwards 1928), five species were described by Brunetti (Brunetti 1911, Brunetti 1912, Brunetti 1918) among them, *Tipula brevis* Brunetti, 1918 was synonymised by Edwards (Edwards 1924). One species was described by Walker (Walker 1848), and one by Savchenko (Savchenko 1956). All the other 28 species were described by Alexander (Alexander 1927, Alexander 1936, Alexander 1942, Alexander 1953, Alexander 1961a, Alexander 1961b, Alexander 1964, Alexander 1968, Alexander 1970, Alexander 1971, Alexander 1973). *Tipula subreposita* Alexander, 1942, was synonymised (Alexander 1963).

Most of Alexander’s species from India were collected by his colleague dr. Fernand Schmid who had collected insect specimens in India and adjoining countries between 1953-1954 and 1958-1960 as a member of the Swiss Zoological Expedition. Amongst Schmid’s entomological collection there is a very rich collection of crane flies, which is presently preserved in C. P. Alexander collection (USNM) (Alexander 1962, Alexander 1966).

**Figure 1.**
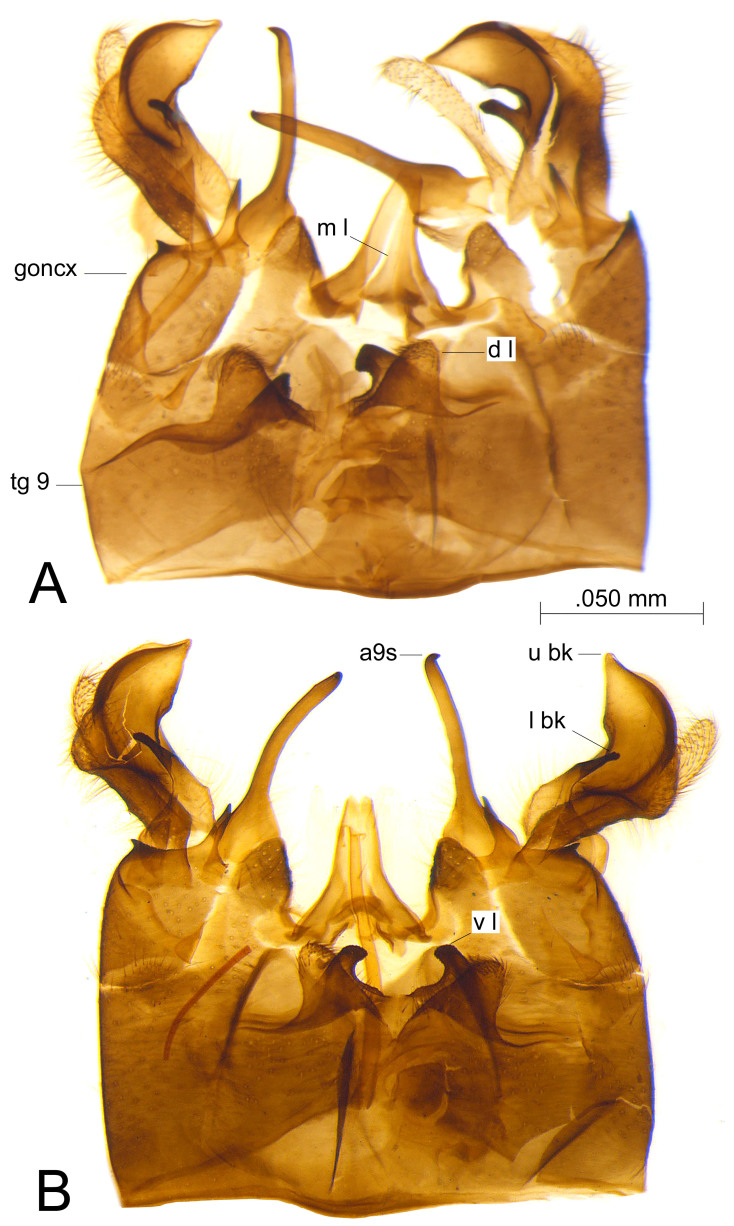
A. *Tipula (V.) wahlgrenana* Alexander, 1968 (holotype) B. *Tipula (V.) hugueniniana* Alexander, 1971 (holotype). Hypopygium, dorsal view. Abbreviations: **a9s** = appendage of sternite 9, **d l, v l** = dorsal and ventral lobe of tergite 9, **goncx** = gonocoxite, **l bk, u bk** = lower and upper beak of inner gonostylus, **m l** = median lobe of adminiculum, **tg 9** = tergite 9.

**Figure 2.**
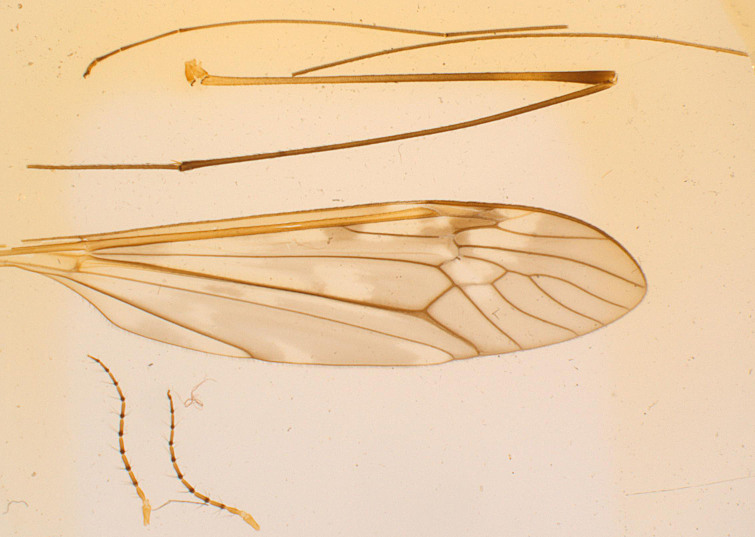
*Tipula (V.) wahlgrenana* Alexander, 1968 (holotype). Antennae, leg and wing on slide mounted (USNM).

*Tipula (V.) wahlgrenana* Alexander, 1968 (Figs 1, 2) was described from single male specimen collected in Kumaon Region by F. Schmid at the end of May of 1958 during his expedition to India. This is the only known specimen representing that species (Alexander 1968).

**Figure 3.**
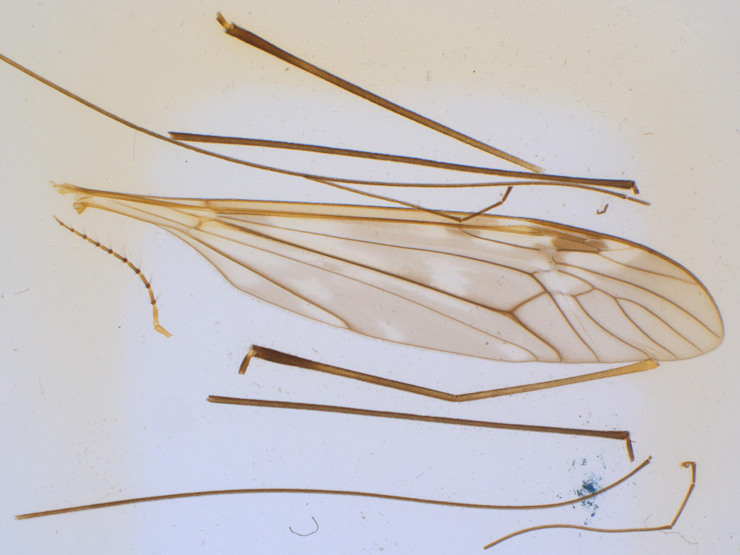
*Tipula (V.) hugueniniana* Alexander, 1971 (holotype). Antenna, legs and wing on slide mounted (USNM).

*Tipula (V.) hugueniniana* Alexander, 1971 (Figs 1, 3) was described from two male specimens also collected by F. Schmid. One male was collected at the same locality only five days later as the holotype of *T. (V.) wahlgrenana*, the other male was collected somewhat east of the previous locality, but two weeks earlier in the following year. These two males are also the only known specimens for the described species (Alexander 1971).

The types of both species were examined. It was found that all the specimens are very similar. In the original description of *Tipula (V.) hugueniniana*, Alexander (1971) mentioned that this species was closely related to *Tipula (V.) gandharva* Alexander, 1951 and *Tipula (V.) tuta* Alexander, 1936, without mentioning its similarity to *Tipula (V.) wahlgrenana*. After detailed analysis of the two holotype and one paratype specimens of *Tipula (V.) wahlgrenana* and *Tipula (V.) hugueniniana*, we found that they cannot be distinguished from each other positively; differences concern only quantitative characters, without qualitative differences. The original description of the morphological details of *Tipula (V.) hugueniniana* in essence repeats the morphological characters of *Tipula (V.) wahlgrenana*. The only difference, according to Alexander’s descriptions, is the general coloration of the mesonotal prescutum, which is obscure yellow in *Tipula (V.) hugueniniana* and light grey in *Tipula (V.) wahlgrenana*. Based on our observation, the coloration of the prescutum varies depending on the angle at which light strikes its surface.

The new synonymy is based on the structure of the male genitalia. All the three examined specimens have the hypopygium as in Fig. 1. Tergite 9 has the posterior border of the dorsal tergal lobes prolonged medially into rounded plates provided with short setulae. The ventral lobes are developed posteriorly into small narrow plates which have a black microscopically scabrous apex. Sternite 9 has a long and slender appendage which has a swollen base and an acute black apex. The gonocoxite has two acute blackened spines, the outer one is small and the inner one is long and slender. The outer gonostylus is an elongate lobe with abundant setae. The inner gonostylus has a small prolonged blackened lobe as lower beak and a massive upper beak. The adminiculum has its median lobe well developed.

Female unknown.

## Supplementary Material

XML Treatment for Tipula (Vestiplex) wahlgrenana
